# Description of the unknown male of *Vappolotestianjiayu* Li, Zhao & Li, 2023 (Araneae, Agelenidae, Coelotinae) from China

**DOI:** 10.3897/BDJ.11.e114147

**Published:** 2023-11-29

**Authors:** Bing Li, Zhe Zhao, Shuqiang Li, Chunyan Xie

**Affiliations:** 1 Hebei Key Laboratory of Animal Diversity, College of Life Sciences, Langfang Normal University, Langfang, Hebei, China Hebei Key Laboratory of Animal Diversity, College of Life Sciences, Langfang Normal University, Langfang Hebei China; 2 Institute of Zoology, Chinese Academy of Sciences, Beijing, China Institute of Zoology, Chinese Academy of Sciences Beijing China; 3 University of Chinese Academy of Sciences, Beijing, China University of Chinese Academy of Sciences Beijing China

**Keywords:** Asia, Wuling Mountains, cave-dwelling spiders, taxonomy

## Abstract

**Background:**

*Vappolotes* Zhao & Li, 2019 is one of the troglophilous genera, with five known species. The previous description of *V.tianjiayu* from China was based solely on female specimens collected from caves in the Wuling Mountains in southern China without any males.

**New information:**

The present study, deals with the first record of the male of *V.tianjiayu* from its type locality: Guluo Cave. The validation of species is based on the morphological characteristics of both male and female.

## Introduction

The changes in ancient oceans (Zhao et al. 2022) and mountains (Li et al. 2021, Yao et al. 2021, Lu et al. 2022) made Asia a region with a high spider diversity. Intensified research on Chinese spiders has increased in recent years and many new species have been discovered and described in China (Li 2020, Liu et al. 2022, Lin et al. 2023). The overwhelming majority of them are found from caves in China (Liu and Li 2013, Lin and Li 2014, Li and Wang 2017, Yang et al. 2023, Yang et al. 2023). The family Agelenidae C.L. Koch, 1837 (Koch 1837) is the tenth largest spider family in the world with 96 genera and 1387 species (WSC 2023). Many species are troglophilous, especially within subfamily Coelotinae, such as *Guilotes* Zhao & Li, 2018 (Li et al. 2018), *Troglocoelotes* Zhao & Li, 2019 (Li et al. 2019a), *Yunguirius* Li, Zhao & Li, 2023 (Li et al. 2023b) and also *Vappolotes* Zhao & Li, 2019 (Li et al. 2019b).

The genus *Vappolotes* is one of the cave-dwelling genera within this subfamily Coelotinae F. O. Pickard-Cambridge, 1893 (Pickard-Cambridge 1893) and comprises five known species: *V.ganlongensis* Zhao & Li, 2019 from Chenjia Cave, *V.hei* Li, Zhao & Li, 2023 from Li Bai Reading Cave, *V.jianpingensis* Zhao & Li, 2019 from Xinlong Cave, *V.longshan* Li, Zhao & Li, 2023 from Da Cave and more caves and *V.tianjiayu* Li, Zhao & Li, 2023 from three unnamed caves and Guluo Cave (Li et al. 2019b, Li et al. 2023a).

After a seven-year interval, two male specimens of *V.tianjiayu* were collected from the previously explored Guluo Cave (type locality), followed by ten females. The long and thin embolus of the male palp matches the long and spiral tubular spermatheca of the female vulva and the strong conductor of the male palp matches the broad copulatory opening of the female epigyne and pocket-shaped copulatory duct of the female vulva. By the collection of female and male specimens together and the matched morphological characteristics, we believe them to be conspecific and present comprehensive descriptions along with accompanying morphological photographs in this study.

## Materials and methods

All examined specimens studied in this paper are deposited in the Institute of Zoology, Chinese Academy of Sciences (IZCAS). Specimens were examined with a LEICA M205 C stereomicroscope at IZCAS. Laboratory habitus photographs were taken with a Sony A7RIV digital camera, equipped with a Sony FE 90 mm Goss lens. Left male palp and epigyne photos were taken with an Olympus C7070 wide zoom digital camera (7.1 megapixels) mounted on an Olympus BX51 compound microscope. Images from multiple focal ranges were combined using Helicon Focus v.6.80 photo stacking software. One left male palp was dissected for examination. Images of it are illustrated. Measurements were obtained with a LEICA M205 C stereomicroscope and are given in mm. Eye diameters were measured as the maximum distance in either dorsal or frontal views. Leg measurements are given as follows: total length (coxa, trochanter, femur, patella, tibia, metatarsus and tarsus). Terminology follows Li et al. (2023b).

Abbreviations of eyes used in the text are as follows: **ALE** anterior lateral eye; **ALE–PLE** distance between ALE and PLE; **AME** anterior median eye; **AME–ALE** distance between AME and ALE; **AME–AME** distance between AME and AME; **AME–PME** distance between AME and PME; **PLE** posterior lateral eye; **PME** posterior median eye; **PME–PLE** distance between PME and PLE; **PME–PME** distance between PME and PME.

## Taxon treatments

### Vappolotes tianjiayu

Li, Zhao & Li, 2023

859D7712-B8AB-55E6-91AD-C389070CA404

urn:lsid:zoobank.org:act:FD59BB0A-7E3B-4CBE-B406-1D3AB94ECE7E

*Vappolotestianjiayu* Li, Zhao & Li, 2023: 330, figs. 4A–E (female).

#### Materials

- **Type status:** Holotype. **Occurrence:** catalogNumber: IZCAS-Ar44376; recordedBy: Yulong Li & Zhigang Chen; individualCount: 1; sex: female; lifeStage: adult; occurrenceID: D45D8BC1-932D-545E-B7D9-0A3BABF36E8E; **Taxon:** scientificName: *Vappolotestianjiayu*; **Location:** country: China; stateProvince: Hunan; locality: Changde City, Shimen County, Hupingshan Town, Tianjiayu Village, an unnamed cave; verbatimElevation: 448 m; verbatimLatitude: 29.9065; verbatimLongitude: 110.8087; **Event:** year: 2016; month: 4; day: 14
- **Type status:** Paratype. **Occurrence:** catalogNumber: IZCAS-Ar44382; recordedBy: Yulong Li & Zhigang Chen; individualCount: 1; sex: female; lifeStage: adult; occurrenceID: 8AC445B4-B138-5E5C-95DB-C02D0E1915F2; **Taxon:** scientificName: *Vappolotestianjiayu*; **Location:** country: China; stateProvince: Hunan; locality: Changde City, Shimen County, Hupingshan Town, Qingshanxi Village, an unnamed cave; verbatimElevation: 442 m; verbatimLatitude: 29.9869; verbatimLongitude: 110.863; **Event:** year: 2016; month: 4; day: 12
- **Type status:** Paratype. **Occurrence:** catalogNumber: IZCAS-Ar44377–381; recordedBy: Yulong Li & Zhigang Chen; individualCount: 5; sex: female; lifeStage: adult; occurrenceID: 51518BFF-8FF2-5431-98B2-7F4E5F3DF325; **Taxon:** scientificName: *Vappolotestianjiayu*; **Location:** country: China; stateProvince: Hunan; locality: Changde City, Shimen County, Hupingshan Town, Tianjiayu Village, an unnamed cave; verbatimElevation: 448 m; verbatimLatitude: 29.9065; verbatimLongitude: 110.8087; **Event:** year: 2016; month: 4; day: 14
- **Type status:** Paratype. **Occurrence:** catalogNumber: IZCAS-Ar44383; recordedBy: Yulong Li & Zhigang Chen; individualCount: 1; sex: female; lifeStage: adult; occurrenceID: A521E119-2755-5FE1-A792-BCDD330D6E65; **Taxon:** scientificName: *Vappolotestianjiayu*; **Location:** country: China; stateProvince: Hunan; locality: Changde City, Shimen County, Hupingshan Town, Tianjiayu Village, another an unnamed cave; verbatimElevation: 455 m; verbatimLatitude: 29.9066; verbatimLongitude: 110.8088; **Event:** year: 2016; month: 4; day: 14
- **Type status:** Paratype. **Occurrence:** catalogNumber: IZCAS-Ar44384–386; recordedBy: Yulong Li & Zhigang Chen; individualCount: 3; sex: female; lifeStage: adult; occurrenceID: 852DEAEF-60CE-5F0F-A8C9-E09087DC0BDD; **Taxon:** scientificName: *Vappolotestianjiayu*; **Location:** country: China; stateProvince: Hunan; locality: Changde City, Shimen County, Hupingshan Town, Guluo Village, Guluo Cave; verbatimElevation: 395 m; verbatimLatitude: 29.9362; verbatimLongitude: 110.8574; **Event:** year: 2016; month: 4; day: 12
- **Type status:** Other material. **Occurrence:** catalogNumber: IZCAS-Ar44788; recordedBy: Zhe Zhao & Xiaoqing Zhang; individualCount: 1; sex: male; lifeStage: adult; occurrenceID: BF5CC0D0-72D0-53D0-9E3F-8D01DB3EEC40; **Taxon:** scientificName: *Vappolotestianjiayu*; **Location:** country: China; stateProvince: Hunan; locality: Changde City, Shimen County, Hupingshan Town, Guluo Village, Guluo Cave; verbatimElevation: 356 m; verbatimLatitude: 29.9389; verbatimLongitude: 110.8523; **Event:** year: 2023; month: 8; day: 29
- **Type status:** Other material. **Occurrence:** catalogNumber: IZCAS-Ar44789; recordedBy: Zhe Zhao & Xiaoqing Zhang; individualCount: 8; sex: female; lifeStage: adult; occurrenceID: 51227165-C745-586E-A5A8-A5CC94248633; **Taxon:** scientificName: *Vappolotestianjiayu*; **Location:** country: China; stateProvince: Hunan; locality: Changde City, Shimen County, Hupingshan Town, Guluo Village, Guluo Cave; verbatimElevation: 356 m; verbatimLatitude: 29.9389; verbatimLongitude: 110.8523; **Event:** year: 2023; month: 8; day: 29

#### Description

**Figure 1.**
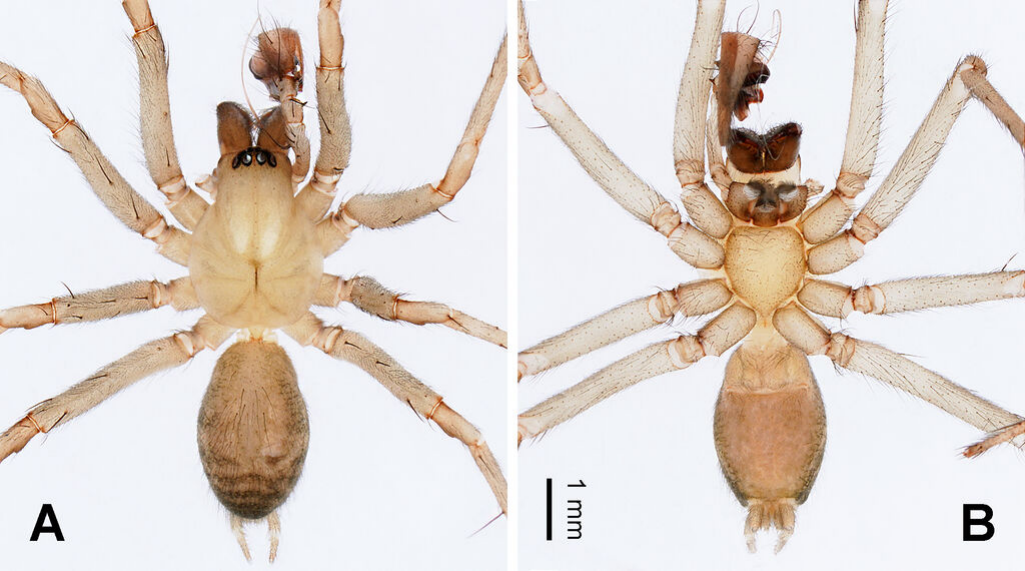
Male habitus of *Vappolotestianjiayu*. **A** dorsal view; **B** ventral view. Scale bar equal for A and B.

**Figure 2.**
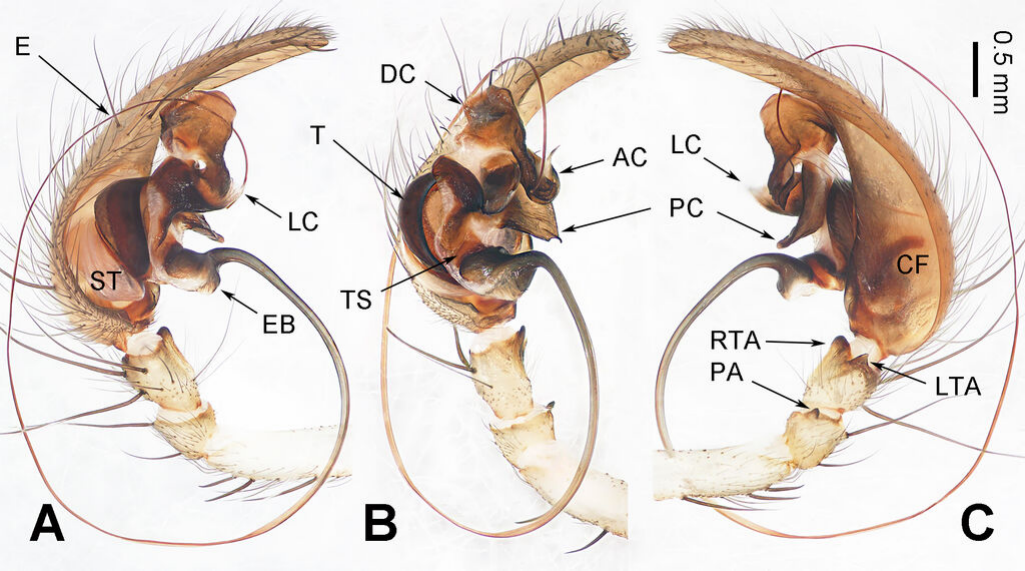
Left male palp of *Vappolotestianjiayu*. **A** prolateral view; **B** ventral view; **C** retrolateral view. Scale bar equal for A–C. Abbreviations: AC = anterior conductor; CF = cymbial furrow; DC = dorsal conductor; E = embolus; EB = embolic base; LC = lamella of conductor; LTA = lateral tibial apophysis; PA = patellar apophysis; PC = posterior conductor; RTA = retrolateral tibial apophysis; ST = subtegulum; T = tegulum; TS = tegulum sclerite.

**Male** (first description) (IZCAS-Ar44788) (Fig. 1A and B). Total length 6.27. Carapace 3.18 long, 2.09 wide. Abdomen 3.09 long, 1.82 wide. Eye sizes and interdistances: AME 0.12, ALE 0.17, PME 0.20, PLE 0.19; AME–AME 0.03, PME–PME 0.07, AME–PME 0.06, ALE–PLE 0.01, AME–ALE 0.03, PME–PLE 0.05. Leg measurements: I: 13.48 (0.83, 0.24, 3.13, 1.11, 3.01, 2.99, 2.17); II: 12.64 (0.88, 0.23, 3.05, 0.94, 2.81, 2.79, 1.94); III: 11.88 (0.81, 0.27, 2.59, 0.98, 2.51, 2.88, 1.84); IV: 15.37 (1.01, 0.34, 3.38, 0.95, 3.38, 4.04, 2.27). Leg formula 4 > 1 > 2 > 3. Carapace yellowish-brown (Fig. 1A), chelicerae reddish-brown with three promarginal teeth and two retromarginal teeth, endites and labium white to brownish (lighter than chelicerae) towards the base (Fig. 1B), sternum yellowish-brown (Fig. 1B), legs yellowish-brown without ring flecks, abdomen grey, with four to five grey chevrons posterodorsally (Fig. 1A), spinnerets yellowish-brown with grey hair (Fig. 1A, B). Palp (Fig. 2A–C): femur long, ca. 3.5 times longer than patella and three times longer than tibia (Fig. 2A); cymbial furrow long and thick, throughout the cymbium (Fig. 2C); patellar apophysis small, ca. 1/6 the length of patella, longer than wide, dark distally (Fig. 2C); retrolateral tibial apophysis thumb-shaped, while lateral tibial apophysis triangular (Fig. 2C); conductor complex, anteriorly spoon-shaped with lamella, dorsally strong and thick with a blunt distal end (Fig. 2B), posteriorly lamellar, with a small concavity, extending diagonally downwards in the retrolateral view (Fig. 2C); embolus long, slender and filiform (Fig. 2A–C), originating around a 3 o’clock position in the ventral view (Fig. 2B).

**Figure 3.**
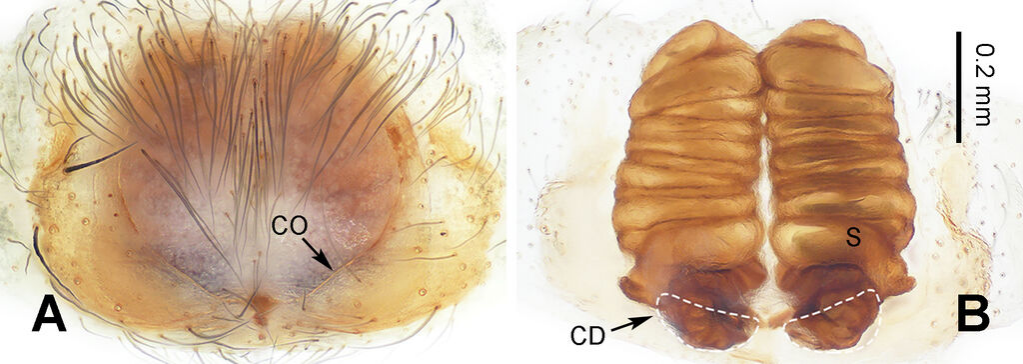
Epigyne of *Vappolotestianjiayu*. **A** epigyne, ventral view; **B** vulva, dorsal view. Scale bar equal for A and B. Abbreviations: CD = copulatory duct; CO = copulatory opening; S = spermatheca.

**Female** (holotype, examined) (IZCAS-Ar44376, XQ270) (Fig. 3A and B cited figs. 4A and B in Li et al. (2023a)). For descriptions, see Li et al. (2023a).

#### Natural History

**Figure 4.**
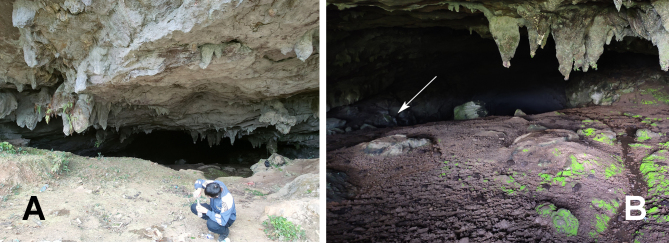
Habitat of Guluo Cave, Hunan, China. **A** entrance to the Guluo Cave (by Cuncun Yang); **B** inside of the Guluo Cave and the white arrow shows where the species inhabit (by Zhe Zhao).

Guluo Cave (entrance to the Guluo Cave in Fig. 4A) is one of the karst caves. Specimens were collected on 29 Aug 2023 from the rock walls of this cave (pointed by the white arrow in Fig. 4B). Previous studies have also documented this species as cave-dwelling species found in karst caves (Li et al. 2023a).

#### Diagnosis

The male *Vappolotestianjiayu* resembles *V.ganlongensis* (type species of *Vappolotes*) by the size and shape of patellar apophysis, retrolateral tibial apophysis and lateral tibial apophysis, long and filiform embolus and spoon-shaped anterior conductor with lamella. However, it can be distinguished from *V.ganlongensis* as follows: 1) anterior conductor accounts for a third of the whole in *V.tianjiayu* (Fig. 2B) vs. accounts for two-thirds of conductor in *V.ganlongensis* (fig. 2B Li et al. (2019b)); 2) dorsal conductor with a blunt distal end in *V.tianjiayu* (Fig. 2B) vs. pointed in *V.ganlongensis* (fig. 2B in Li et al. (2019b)); 3) embolic base beginning horizontally in *V.tianjiayu* (Fig. 2A–C) vs. vertically in *V.ganlongensis* (fig. 2A–C in Li et al. (2019b)). For diagnosis of the female, see Li et al. (2023a).

#### Distribution

China, Hunan (Li et al. 2019b, Li et al. 2023a and current record).

## Supplementary Material

XML Treatment for Vappolotes tianjiayu
